# Resequencing and De Novo Assembly of *Leishmania* (*Viannia*) *guyanensis* from Amazon Region: Genome Assessment, Phylogenetic Insights and Therapeutic Targets

**DOI:** 10.3390/pathogens15010124

**Published:** 2026-01-22

**Authors:** Lucas George Assunção Costa, Edivaldo Costa Sousa Junior, Camila Cristina Cardoso, Millena Arnaud Franco da Igreja, Franklyn Samudio Acosta, Fabiano Reis da Silva, Lourdes Maria Garcez

**Affiliations:** 1 Laboratório de Epidemiologia das Leishmanioses, Seção de Parasitologia, Instituto Evandro Chagas, Rodovia BR 316, km 07, s/n, Ananindeua 67.030-000, PA, Brazil; lucas.27@hotmail.com (L.G.A.C.); edivaldojunior@iec.gov.br (E.C.S.J.); camilaccg94@gmail.com (C.C.C.); millena.afdigreja@gmail.com (M.A.F.d.I.); fabianobiologico@gmail.com (F.R.d.S.); 2 Instituto Conmemorativo Gorgas de Estudios de la Salud, Avenida Justo Arosemena, Panama City P.O. Box 0816-02593, Panama; franklynsamudio@hotmail.com; 3 Facultad de Ciencias Naturales Exactas y Tecnología, Universidad de Panamá, Panama City P.O. Box 0824-00073, Panama; 4 Departamento de Patologia, Centro de Ciências Biológicas e da Saúde, Universidade do Estado do Pará, Travessa Perebebui, 2623, Belém 66.087-662, PA, Brazil

**Keywords:** *Leishmania guyanensis*, genome assembly, genomic annotation, *polA1*, phylogeny, orthologous genes

## Abstract

*Leishmania guyanensis* is one of 15 American human-pathogenic species, frequently linked to therapeutic failure due to its marked genetic plasticity and adaptability under drug pressure. To broaden the genomic understanding of this species, its biological traits, and potential therapeutic alternatives, we sequenced the *L. guyanensis* strain MHOM/BR/75/M4147. Raw reads underwent quality-filtering and assembly. Taxonomic classification utilized BLASTn and Kraken2, confirming that 99.95% of contigs matched *Leishmania*. The assembled genome size was 31 Mb, with an N50 of 4743 bp and 40.85× coverage. Variant calling subsequently identified 36,665 SNPs, 8210 indels, and chromosomal aneuploidies. Genomic annotation identified 3119 proteins with known molecular functions in *L. guyanensis*, alongside 6371 orthologous genes shared with *L. major* and *L. panamensis*. The search for pharmacological relevance yielded ten candidate genes, including one calpain and nine GSK3 family members. Phylogenetic reconstruction using the *polA1* gene consistently grouped *L. guyanensis*, demonstrating strong discriminatory capacity, with *L. martiniquensis* emerging as the most divergent species. Overall, these findings expand the available genomic framework for *L. guyanensis* and support advances in species-specific diagnostic approaches.

## 1. Introduction

The *Leishmania* genus (Kinetoplastida; Trypanosomatidae) includes species responsible for a spectrum of tropical zooanthroponotic diseases known as leishmaniasis. The common clinical manifestations of the disease are cutaneous leishmaniasis (CL), mucocutaneous leishmaniasis (ML), and visceral leishmaniasis (VL). The protozoans of this genus are transmitted to mammal reservoirs, including humans, by the bite of several phlebotomous species (Psychodidadae: Phlebotominae), which are broadly spread in forest areas [1,2,3].

The diverse species of the *Leishmania* genus cluster in four subgenera: *Leishmania* (*Viannia*), *Leishmania* (*Leishmania*), *Leishmania* (*Sauroleishmania*), and *Leishmania* (*Mundinia*). The first subgenus includes *Leishmania* pathogens broadly distributed in the Americas. Nearly fifty *Leishmania* species are currently known; twenty are human pathogens, including 15 endemics in the Americas [4,5]. The *Leishmania* (*Viannia*) subgenus is characterized by its development in the hindgut of the sandfly vector (peripylarian development), a distinct feature from the *Leishmania* (*Leishmania*) subgenus (suprapylarian development) [6]. While *Leishmania* (*Viannia*) species, such as *L*. *braziliensis* and *L*. *guyanensis*, are the primary agents of cutaneous and mucocutaneous leishmaniasis in the Neotropics, they remain less familiar to some international clinical settings compared to the *Leishmania* (*Leishmania*) subgenus, which has a broader global distribution including both the Old and New World. Consequently, it is mandatory to carry out studies on the comparative genomics of the species housed in this subgenus that may reveal differences useful to explain how biological traits of *Leishmania* (*Viannia*) parasites impact medically relevant characteristics such as drug resistance, clinical outcome, virulence, and pathogenicity.

Formerly, CL and ML complications were commonly associated with *L.* (*V*.) *braziliensis*, the most disseminated species in the Americas. However, it is currently known that *L.* (*V.*) *guyanensis* causes not only single skin lesions (or multiple lesions at a determined frequency) but also nasopharyngeal mucosa destruction in more severe cases [7,8]. This species occurs in several Latin American countries such as Panama, Brazil, Bolivia, Colombia, French Guiana, Suriname, Venezuela, Ecuador, and Argentina [9,10,11]. *Leishmania* (*Viannia*) *guyanensis* transmission is strongly related to human displacement through heavy vegetation where the presence of mammal species such as *Choleopus didactylus*, *Tamandua tetradactyla*, and *Didelphis marsupialis*, natural reservoirs of this parasite, occurs. In Brazil, the *L*. (*V*.) *guyanensis* distribution is especially broad in the Amazon region, where drug failure rates are high, and a different treatment choice is mandatory due to the predominance of this *Leishmania* species [12,13].

Under pressure, *L.* (*V.*) *guyanensis* develops adaptive mechanisms capable of inducing drug uptake/efflux modifications in the first-line therapy used to treat CL, which causes treatment failure [14,15]. These and other biological characteristics, including virulence, pathogenicity, proliferative capacity, and immune modulation, are directly related to pathogens’ genomes [16].

Genomic plasticity is a common feature of the *Leishmania* genus, including aneuploidy and chromosomal fission/fusion events. Moreover, the kinetoplastid genome organization shows genes grouped in polycistronic transcription units and an evident scarcity of introns in their genes. This fact evidences a basal genomic structure without most of the more complex modular genetic elements found in other eukaryotes [17,18]. The *L.* (*V.*) *guyanensis* genome has an average size of 32 Mb, containing nearly 8000 protein-coding genes and 35 chromosomes due to the fusion of chromosomes 20 and 34 [16]. When thoroughly described, this genomic data represents a source of accurate and robust information necessary for understanding parasite adaptability and survival strategies that might contribute to developing efficient therapeutic interventions.

In the middle of several initiatives aimed at sequencing genomes of parasites from the *Leishmania* genus [16,19,20], this study describes the genome of a *L*. (*V*.) *guyanensis* strain from the Amazon, a *Leishmania* species poorly investigated [21].

This study aimed to perform the resequencing and de novo assembly of the *Leishmania* (*Viannia*) *guyanensis* strain MHOM/BR/75/M4147, followed by comprehensive genomic annotation to characterize its functional architecture and identify potential therapeutic targets. Despite the epidemiological importance of *L. guyanensis* in the Americas, high-quality reference genomes for this species remain scarce, limiting comparative genomics and the identification of species-specific markers. To address this gap, we also evaluated the *polA1* gene as a phylogenetic marker to assess its ability to discriminate species and complexes within the *Viannia* subgenus. Together, these analyses provide a robust genomic baseline to support improved diagnostics, targeted therapies, and future molecular and epidemiological studies of cutaneous and mucocutaneous leishmaniasis.

## 2. Materials and Methods

### 2.1. Ethics

This study does not involve human subjects or tissue samples but only *Leishmania* promastigotes and computational analyses; therefore, ethical approval is not required.

### 2.2. DNA Extraction and Sequencing

The *Leishmania guyanensis* strain was provided by a *Leishmania* Collection (CLIOC) that is part of the Network of Biological Resource Centers for Conformity Assessment of Biological Material, Instituto Oswaldo Cruz, Rio de Janeiro, Brazil. The genomic DNA of the *L.* (*V*.) *guyanensis* strain (MHOM/BR/75/M4147) was isolated using the Wizard^®^ Genomic DNA Purification kit (Promega, Madison, WI, USA) following the manufacturer’s instructions, rehydrating the whole DNA in a final volume of 50 µL. The genomic library of this parasite was then prepared using the Nextera XT DNA library preparation kit (Illumina, Inc., San Diego, CA, USA) following the manufacturer’s protocols to subsequently be sequenced by the NextSeq 500 (Illumina, Inc., San Diego, CA, USA) platform using the NextSeq 500/550 High Output Kit v2.5 (300 cycles) and a paired-end sequencing approach.

### 2.3. Reads Quality Assessment

To get rid of adapters and low-quality reads (Phred < 20) after the sequencing process, we used the Fastp software v.0.20.1 [22] and the software FastQC v.0.12.1 [23] to depict read-quality data.

### 2.4. Genome Assembly

We used a de novo assembly approach employing no reference genome to assemble all reads in contigs using the MEGAHIT v.1.2.9 assembly software. This assembler utilizes a succinct Bruijn Graph (SdBG) and an iterative multi k-mer strategy, which efficiently handles large datasets and improves the recovery of low-coverage and repetitive regions [24]. To create scaffolds from the assembled contigs, we used the software SSPACE v.3.0 [25].

### 2.5. Taxonomical Classification of Reads and Contigs

Assembled contigs resolve repetitive regions of the complex *Leishmania* genome, minimizing ambiguities that raw reads cannot solve alone. This provides the long consensus sequences necessary for accurate gene annotation and species differentiation. To perform the taxonomical classification of reads and contigs obtained, we first infer the sequence homology of these sequences by a local alignment with sequences from a local *Leishmania* DNA database (Supplementary Material S1) using the BLASTn algorithm v.2.5.0 [26]. After sequence homology determination, we inferred the taxonomical status of reads and contigs by performing a classification based on k-mer and adding taxonomical tags to each sequence using Kraken2 software v.2.1.2 [27] with the PlusPFP-16 database, which contains RefSeq sequences for archaea, bacteria, viruses, humans, protozoa, fungi, plants, and the UniVec library for screening synthetic vector contaminants. For the visualization and analysis of the contig classification data, the Pavian v.1.0. application was employed [28]. This classification allowed us to extract all contigs belonging to *Leishmania* to perform the genomic annotation of *L*. (*V*.) *guyanensis* (MHOM/BR/75/M4147) subsequently.

### 2.6. Genome Annotation

The genomic annotation of this parasite was performed using the AUGUSTUS software v.3.5 [29], using as a reference the genomic structure of *Leishmania* (*Sauroleishmania*) *tarentolae*. We constructed a local genomic database of 69 *Leishmania* genomes (Supplementary Material S1) retrieved from GenBank to compare genomic annotations and analyze and correct open reading frames (ORFs) positions and structures. In this way, we provided more accurate evidence of gene function during the gene annotation of this parasite. To depict the genome and manually edit ORFs obtained during the annotation process, we used the bioinformatics suite Geneious v.8.1.4 [30]. The manual edition was finished when we analyzed the base quality and integrity of each ORF.

### 2.7. Gene Orthologs Assessment

The genomic data of *L*. (*V*.) *guyanensis* (MHOM/BR/75/M4147) was used to perform a Pangenome analysis aimed at identifying ortholog genes comparing this genome to the genome assembly of *Leishmania* (*V.*) *shawi* (GCA_962240455), *Leishmania* (*V*.) *panamensis* (GFC_000755165.1) and *Leishmania* (*L*.) *major* (GCF_000002725.2). To do so, we used the software BLASTp v2.5.0 [31] and the statistical software R v.4.1.2. using the Venn library [32].

### 2.8. Drug Target Identification

A structure-guided homology-based strategy was employed to screen for candidate drug targets associated with leishmaniasis. For this purpose, a curated local reference dataset was assembled comprising amino acid sequences derived from experimentally resolved three-dimensional protein structures with established relevance in antiparasitic drug research. These structures, previously explored in ongoing studies conducted in our laboratory (unpublished data), were obtained from the RCSB Protein Data Bank and included representatives of key molecular targets such as calpains (PDB IDs: 1TLO, 1MDW, 1ZCN), the dual-specificity kinase DYRK1A (3ANQ), glycogen synthase kinase 3 (GSK3; 7S6V), and an ATP-binding cassette (ABC) transporter (7OJ8). Predicted protein sequences from the analyzed genomes were queried against this reference dataset using BLASTp from the BLAST+ package (version 2.15.0) [33], with the aim of detecting conserved functional domains indicative of pharmacological relevance. To minimize spurious matches and enhance biological interpretability, BLASTp outputs were subjected to a stringent post-processing step. Only alignments that satisfied the following criteria were retained: (i) amino acid identity ≥ 35%, (ii) alignment length ≥ 60 residues, (iii) e-value ≤ 1 × 10^−3^ and (iv) bitscore ≥ 30. These thresholds are widely accepted in the field for detecting functionally relevant homologies with a moderate degree of conservation. These cutoffs are commonly adopted in protein homology analyses to balance sensitivity and specificity when identifying moderately conserved functional relationships [34]. Following filtering, retained matches were prioritized using a composite ranking scheme that integrated multiple alignment attributes, namely percentage identity, alignment coverage, statistical confidence expressed as the logarithm of the e-value, and bitscore. The top 200 highest-ranked hits were selected and subsequently merged into a unified dataset, enabling a global and comparative assessment of candidate drug targets across the genome.

### 2.9. Mapping to Reference Genome, Genome Coverage, and Variant Call

The mapping to the reference genome of *L.* (*V.*) *guyanensis* was performed using Bowtie2 [35], allowing an assessment of chromosomal coverage. This read mapping also allowed us to determine chromosome ploidy (chromosome copy number for each genome) by dividing each chromosome coverage by half of each 35 chromosome’s average coverage and assuming a diploid organism. Single-nucleotide polymorphisms (SNPs) and insertion/deletion (indels) events were identified using BCFtools v1.2 [36] to detect nucleotide differences between the resequencing strain of *L.* (*V.*) *guyanensis* (MHOM/BR/75/M4147) and the reference genome of this species (GenBank CP103914-CP103949).

### 2.10. Phylogenetic Inference

The DNA polymerase alpha catalytic subunit (*polA1*) was selected as the best molecular target to infer *Leishmania* phylogeny after evaluating its phylogenetic signal with the TREE-PUZZEL v.5.3 algorithm [37] using the likelihood-mapping method (Supplementary Material S2). To assess the phylogenetic signal, we used 69 *Leishmania* genomes (Supplementary Material S1). The *polA1* gene from these genomes was identified and extracted using the software Geneious v.8.1.4 to perform a phylogenetic analysis using the maximum likelihood method (ML) included in the IQTREE2 software v.2.0.7 [38] after selecting the best nucleotide substitution model by the algorithm of the software. The software parameters were set up to find the most likely tree topology, testing each clade’s robustness by bootstrap analysis. The choice of the best phylogenetic tree considered the genetic identity threshold among *Leishmania* species inferred by a sequence identity matrix constructed by the software. All software parameters were set to standard conditions unless another configuration was required.

## 3. Results

### 3.1. Quality Control and Genome Assembly

The genome sequencing of the *L.* (*V.*) *guyanensis* (MHOM/BR/75/M4147) produced 9,847,950 reads with an average size of 151 bp. After filtering low-quality reads and excluding adapters and artifacts from the sequencing process, we obtained 8,839,174 high-quality reads, a reduction of 10%. The de novo assembly produced 14,097 contigs with an N50 value of 4743 bp, an average contig size of 2239 bp, and an average genome coverage of 40.85×. The number of scaffolds constructed was identical to the number of contigs (14,097). The genome size of the *L.* (*V.*) *guyanensis* was 31,565,639 bp (31 Mb) with a G + C content of 57.44%. Consequently, we obtained a new complete genome assembly (whole genome shotgun) for the *Leishmania* isolate analyzed herein. Table 1 gathers all the genome assembly results obtained in this study and those produced in earlier *Leishmania* genomic studies. The genome assembly for *L.* (*V.*) *guyanensis* (MHOM/BR/75/M4147) was deposited in the GenBank database under the accession number GCA_051201275.1. The data is publicly available at https://www.ncbi.nlm.nih.gov/datasets/genome/GCA_051201275.1/ (accessed on 12 January 2026).

### 3.2. Taxonomical Assessment

Most of the contigs produced in this study (99.95%, 14,090 contigs) were found to be related to the *Leishmania* genus (Supplementary Material S3). All selected contigs showed an average nucleotide identity value of 96.29% with *Leishmania* spp. The contigs tagged as belonging to *Leishmania* spp. were then used in the genomic annotation process.

### 3.3. Variants Call and Chromosome Coverage

**Figure 1:**
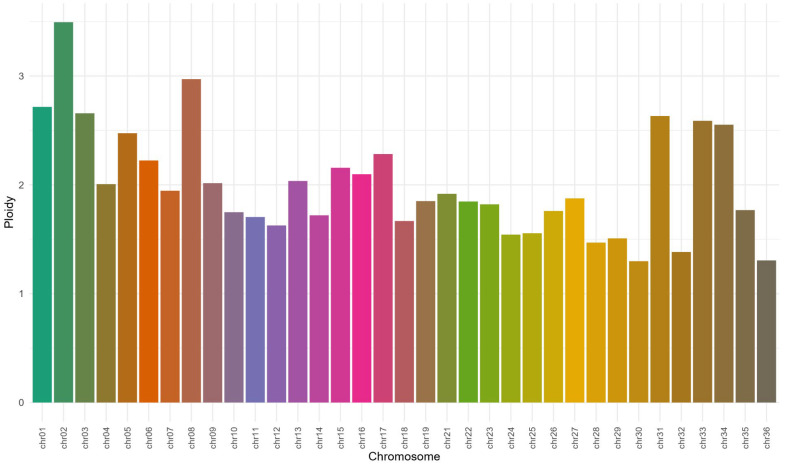
Analysis of chromosomal ploidy in *Leishmania guyanensis* (MHOM/BR/75/M4147) strain, demonstrating aneuploidy. Chromosomal dosage increase is observed, with emphasis on chromosomes 2 and 8, which exhibit conditions approaching trisomy.

We searched for variants and identified 36,665 SNPs distributed through the 35 *L.* (*V.*) *guyanensis* chromosomes and 8210 indels (Supplementary Material S4). Reads mapping to the *L.* (*V.*) *guyanensis* reference genome (GenBank CP103914-CP103949) identified chromosomes with coverage values between 41× and 111× (Supplementary Material S4). Additionally, there was great heterogeneity in the chromosome ploidy (values between 1.2 and 3.4), highlighting chromosomes 2 and 8 that presented a condition close to trisomy. The ploidy variations throughout *L.* (*V.*) *guyanensis* chromosomes are depicted in Figure 1.

### 3.4. Genomic Annotation

**Figure 2:**
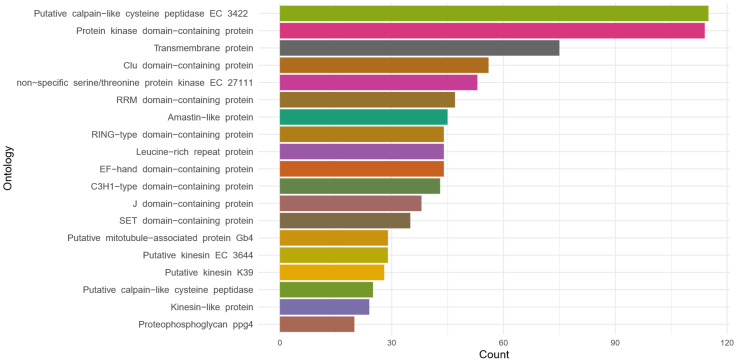
Principal functional proteins identified in the genomic annotation of *Leishmania guyanensis*. The list presents the twenty proteins associated with the highest number of corresponding genes, highlighting functionally relevant components within the species’ genome.

The gene annotation of the *L.* (*V.*) *guyanensis* (MHOM/BR/75/M4147) revealed a lack of introns and allowed us to identify 7698 genes, 93.42% (7192) of which were associated with known molecular functions. After analyzing all the annotated data, we identified 3119 different proteins related to the metabolic pathways of the parasite. The proteins with more associated genes were calpain-like cysteine peptidase (155), protein kinase domain-containing protein (114), and transmembrane protein (75). Figure 2 depicts the main 20 relevant proteins identified during the annotation process.

### 3.5. Gene Orthologs Analysis

**Figure 3:**
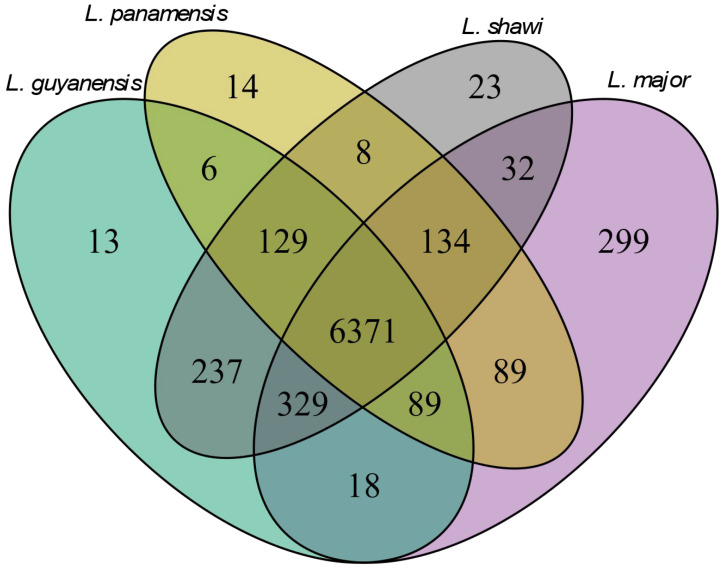
Venn diagram comparing shared genes among *Leishmania major*, *L. panamensis*, *L. guyanensis*, and *L. shawi*. The area overlap indicates the number of genes common to the species, highlighting both species-specific (exclusive) genes and those shared across each group.

The gene annotation of *L.* (*V.*) *guyanensis* (MHOM/BR/75/M4147) allowed us to study the Pangenome, ortholog genes, and core genome that altogether represent the entire group of annotated genes extant in a particular taxonomic group [5]. Therefore, we identified 6371 genes shared by *L.* (*V.*) *guyanensis*, *L.* (*V.*) *panamensis*, *L.* (*V.*) *shawi*, and *L.* (*L.*) *major*. Fourteen of these genes were exclusive to *L.* (*V.*) *panamensis*, 13 of *L.* (*V.*) *guyanensis*, 23 of *L.* (*V.*) *shawi*, and 299 of *L.* (*L.*) *major*. The Pangenome of these four *Leishmania* species encompasses 7791 genes. Regarding the *Leishmania* species core genome, each species showed a characteristic genetic expansion (expansion in ortholog copy numbers). For example, we found 22 expanded genes in *L.* (*V.*) *guyanensis* MHOM/BR/75/M4147 (Supplementary Material S5). The Pangenome analysis result is depicted in Figure 3.

### 3.6. Identification of Therapeutic Targets

Protein homology analysis in *Leishmania* (*Viannia*) *guyanensis* led to the identification of ten candidate genes with potential pharmacological relevance, as summarized in Table 2, with a marked predominance of proteins belonging to the GSK3 family (9/10 genes), in addition to a single representative of the calpain family. The gene g7485.t1_1, annotated as GSK3, exhibited the highest quality score (0.968), accompanied by very high amino acid identity (92.96%), an extensive alignment length (355 residues), and an exceptionally high bitscore (701), indicating strong structural and functional conservation relative to the reference target. Collectively, these parameters support this gene as the most robust pharmacological candidate identified in the analyzed genome.

**Figure 4:**
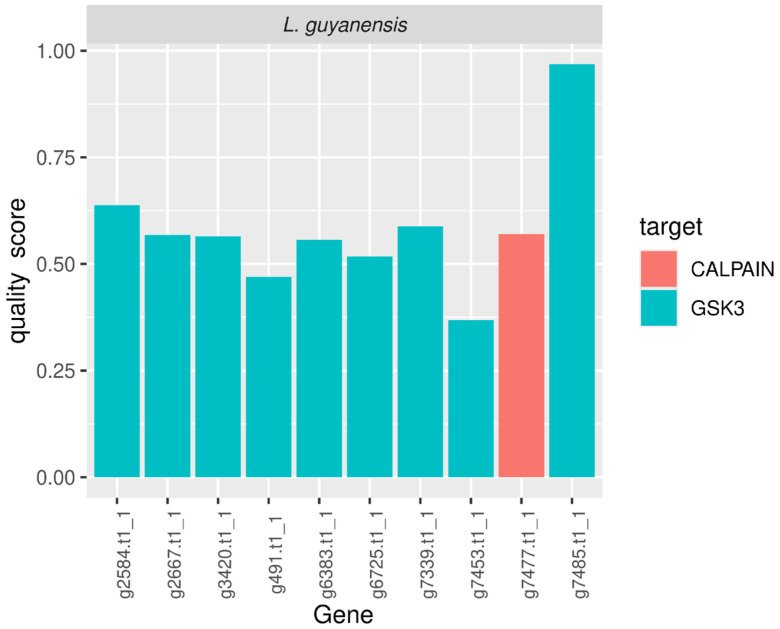
Graphical representation of the normalized quality scores of candidate drug target genes identified in the *L.* (*V.*) *guyanensis* genome. Genes belonging to the GSK3 family exhibit predominantly higher scores, with gene g7485.t1_1 standing out as the most conserved and structurally robust candidate, whereas the remaining genes show intermediate scores associated with partial alignments or moderate sequence identity.

The remaining GSK3-annotated genes displayed intermediate quality scores, ranging from 0.37 to 0.64, corresponding to alignments with moderate amino acid identity (35–53%), alignment lengths between 68 and 135 residues, and highly significant e-values (≤10^−10^). The single calpain-annotated gene (g7477.t1_1) showed a quality score comparable to those of the intermediate GSK3 candidates (0.57), despite a shorter alignment length, suggesting the presence of partially conserved functional domains (detailed results are provided in Table 2). The distribution of quality scores, shown in Figure 4, reveals a clear hierarchical organization of candidate targets, with a strong predominance of GSK3-related proteins, reinforcing this family as the principal set of potential drug targets in *L.* (*V.*) *guyanensis*.

### 3.7. Phylogenetic Tree Reconstruction

**Figure 5:**
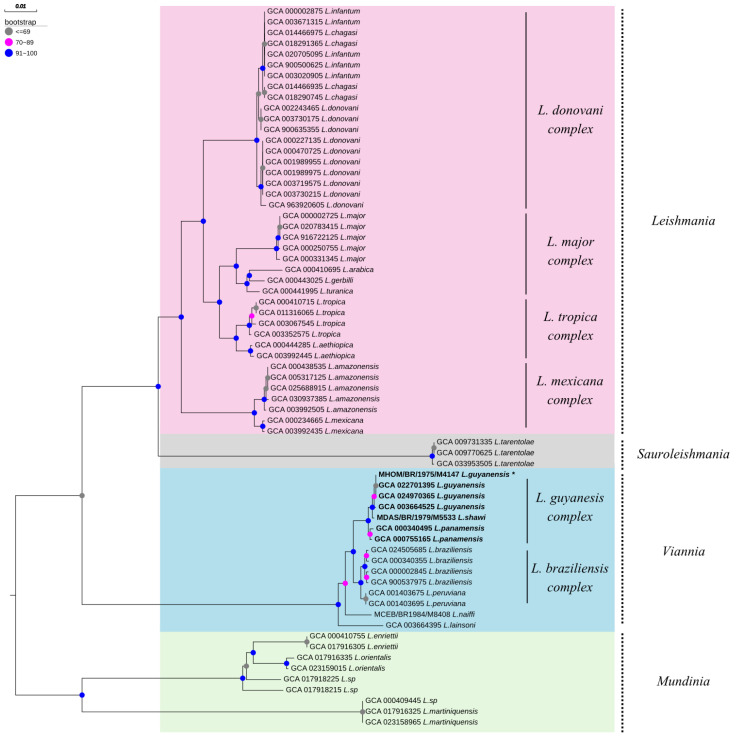
Phylogenetic tree based on the *polA1* gene sequence, illustrating the phylogenetic groupings within the *Leishmania* genus. The tree organizes the species into hierarchical levels, including subgenera, complexes, and species. * MHOM/BR/1975/M4147 *L. guyanensis* refers to the strain sequenced in the present study.

As a result of the phylogenetic analysis, the four Leishmania subgenera were grouped into independent monophyletic clusters supported by bootstrap values higher than 90%. Furthermore, all Leishmania complexes within each subgenus clustered into specific subclades supported by bootstrap values around 90% or higher. Regarding the sequence identity analyses, the species from the subgenera Leishmania (Leishmania), Leishmania (Sauroleishmania), Leishmania (Viannia), and Leishmania (Mundinia) showed sequence identity values of 90%, 99.8%, 96.8% and 84.7%, respectively (Figure 5).

## 4. Discussion

### 4.1. Genome Assembly Integrity and Taxonomic Resolution

The genome assembly of *L.* (*V.*) *guyanensis* (MHOM/BR/75/M4147) performed herein strengthens the extant genomic data of this *Leishmania* species, providing excellent values of completeness and coherence when compared with the current *L.* (*V.*) *guyanensis* reference genome. An N50 value of 6029 bp supported the good quality of the genome assembly as the parameter suggested that most of the contigs were long enough and well-assembled. The clear evidence that the scaffold number obtained was identical to the contigs number also supports the completeness of the assembled contigs, which are long enough and need no additional connections as links or bridges to create scaffolds. This fact reflects the good quality of the genome assembly obtained by the de novo method, a significant advancement considering that other *Leishmania* species of epidemiological importance still lack high-quality genome assembly metrics (Table 1). Certainly, the quality filtering and taxonomical identification of the reads were critical for obtaining the correct genome structure [40], since 9% of the reads were classified as sequencing artifacts.

The reference genome of *L.* (*V.*) *guyanensis* (GenBank CP103914–CP103949) presented an average coverage value of 27.0×, while the coverage value obtained in this study for the same *Leishmania* strain was 40.85×. This higher coverage might have helped to acquire more accurate results in other genome-based analyses, such as SNP identification and variant calls.

As the *L.* (*V.*) *guyanensis* genome assembly performed in this study, the assembly reported earlier for the *L.* (*V.*) *guyanensis* LgCL085 genome [39] presented good assembling parameters. However, a different bioinformatic workflow, including assembly corrections, gap filling, and junction joining, was necessary to complete the genome assembly. Both *L.* (*V.*) *guyanensis* (LgCL085) and *L.* (*V.*) *panamensis* (PSC-1) genomes available in the GenBank were characterized via de novo assembly, representing the only strains from the *L.* (*V.*) *guyanensis* complex with complete genomes available in genome databases. The genomic characterization of *L.* (*V.*) *guyanensis* (MHOM/BR/75/M4147) performed in this study complements the genomic data of this species, providing indicative parameters of quality and completeness lacking in most genomic studies of *Leishmania*. Generally, the genome assembly of *L.* (*V.*) *guyanensis* (MHOM/BR/75/M4147) obtained herein showed similar genomic characteristics as the ones previously reported for *L.* (*V.*) *braziliensis* [41], *Leishmania* (*Leishmania*) *amazonensis* [42], and *L.* (*V.*) *panamensis* [16], which present genome sizes of nearly 30 Mb, average G + C content of 57%, and a value of predicted gene quantity close to 8000. This result indicates convergence among methods used to assemble *Leishmania* genomes, highlighting the importance of resequencing previously characterized *Leishmania* species, especially those with intrinsic epidemiological and clinical relevance.

### 4.2. Chromosomal Aneuploidy and Genomic Plasticity

Aneuploidy, defined by chromosomal copy number variation (CNV), is a widely recognized trait in *Leishmania*, directly associated with the parasite’s adaptive plasticity and resulting in cellular mosaicism [43,44]. Ploidy analysis demonstrated that the resulting karyotypic profile is highly dependent on the reference genome employed for read mapping. When utilizing the homologous *L*. (*V*.) *guyanensis* (M4147) reference (characterized by a continuous and gapless assembly), chromosomes 2 and 8 exhibited values approaching trisomy, suggesting lineage-specific amplification events. In contrast, mapping against the *L*. (*V*.) *braziliensis* (M2904) reference revealed a distinct pattern, with chromosomes 8 and 31 showing supernumerary status.

This discrepancy is particularly relevant for chromosome 31, a recognized evolutionarily conserved copy number variation (CNV) locus recurrently associated with aneuploidy, which has been linked to phenotypic adaptation, including stress responses and gene expression modulation, conferring a potential selective advantage [45,46]. The observed distinctions between ploidy profiles likely reflect the interplay between strain-intrinsic characteristics (possibly influenced by conservation history) and methodological factors associated with quality metrics, architecture, and structural differences between official, despite their phylogenetic proximity, reference genomes. Thus, while the frequent detection of supernumerary chromosome 31 reinforces its constitutively polysomic nature, aneuploidy must be considered a dynamic and genome context-dependent phenomenon in *Leishmania.*

A more significant deviation from the disomic state was observed during the read mapping in chromosomes 2 and 8. This fact also supports that the genomic reference choice significantly affects karyotype even among species belonging to the same subgenus, highlighting the importance of using a specific reference genome from the same species to perform more accurate analyses. Although *L*. (*V*.) *guyanensis* (MHOM/BR/75/M4147) was mapped against its own reference genome, 36,665 SNPs were identified. These variations likely reflect micro-evolutionary changes during independent laboratory cultivation. Notably, as specific information regarding the in vitro passage history of the reference genome was unavailable, this factor, combined with the use of different sequencing technologies, may account for the observed genetic differences. Such variability often arises during parasite culture in response to distinct microenvironments, leading to adaptive genetic shifts that favor survival under varying environmental stimuli [47].

### 4.3. Genomic Annotation and Comparative Orthology

The study carried out herein complements the genetic architecture previously described for *L.* (*V.*) *guyanensis* (MHOM/BR/75/M4147) (reference), identifying genes (7726) and describing functional proteins. In this sense, the gene ontology analyses of the *L.* (*V.*) *guyanensis* (LgCL085) revealed the presence of 8230 protein-coding genes. Most of these proteins were associated with ortholog genes reported in the *Viannia* subgenus whose copy numbers vary from one species to another [39]. Due to the polycistronic nature of this genus, each protein is not strictly associated with one gene, and there is also no coding DNA and ORFs coding for more than one protein. This fact was verified after finding a high level of genes associated with one functional protein. The initial description of 3128 protein-coding genes in *L.* (*V.*) *guyanensis* might aid in disclosing the translational composition of this species, a fact of paramount importance for understanding its cellular and metabolic processes [41].

The gene annotation was of value to identify essential elements, including start and stop codons, exon-intron junctions, protein-coding regions, and promoters and regulatory regions. As a result, we observed a lack of introns in most of the *L.* (*V.*) *guyanensis* protein-coding genes, evidence previously reported in genomic studies of *Leishmania* earlier [48], and that could be linked to inherent polycistronic characteristics of the *Leishmania* genus. This polycistronic region includes a DNA region processed during transcription to produce a long polycistronic RNA including non-related genes organized into a genomic architecture resembling the prokaryotes’ gene organization [49,50].

The workflow described herein identifies proteins and ortholog genes in microorganisms in general and has also been used earlier to assess *Leishmania* spp. Genomes [39]. Using this strategy, we identified ortholog genes shared by *L.* (*V.*) *guyanensis*, *L.* (*V.*) *panamensis*, and *L.* (*L.*) *major* and a reduced group of genes (48) restricted to *L.* (*V.*) *guyanensis*. Among these three species, *L.* (*L.*) *major* showed the highest number of genes unrelated to the other species (1631), probably because it belongs to a different *Leishmania* subgenus. Conversely, we found fewer exclusive genes between *L.* (*V.*) *panamensis* and *L.* (*V.*) *guyanensis*. Generally, gene ortholog studies have indicated low quantities of species-specific genes in *Leishmania* [16,19,36]. Additionally, we observed a trend indicating that closely related *Leishmania* species from the same complex share more ortholog genes in the Pangenome than those *Leishmania* parasites from different subgenus. This might be explained by the great divergency bridge between *Leishmania* spp. from different subgenus due to the parasites’ adaptation to conditions found in each transmission cycle. This biological adjustment of parasites from different *Leishmania* subgenera during the evolutionary process might explain the functional diversity found in the *Leishmania* genus nowadays. Indeed, the functional diversity produced in this way might account for the differential group of orthologs belonging to the different taxonomic units within the *Leishmania* spp.

### 4.4. GSK3 and Calpain as Conserved Therapeutic Targets

The marked predominance of GSK3 family members among the highest-ranked candidate genes in *Leishmania* (*Viannia*) *guyanensis* highlights the central role of this kinase as a highly conserved and biologically relevant pharmacological target in this taxon [51]. The identification of nine GSK3-annotated genes among the ten top-scoring candidates reflects both the robustness of the structure-guided homology-based strategy employed in this study and the functional importance of this protein family in trypanosomatids [52]. GSK3 has been consistently implicated in the regulation of essential cellular processes, including cell-cycle control, intracellular signaling, and parasite adaptation to host-derived stresses, all of which are critical for parasite survival and pathogenicity [53].

Within this context, the gene g7485.t1_1, which exhibited the highest quality score, emerges as a particularly strong candidate for pharmacological exploration. The combination of high amino acid identity, extensive alignment coverage, and exceptional statistical significance suggests the presence of a complete and structurally conserved catalytic domain. Such features are characteristic of essential enzymes and are commonly associated with high-value therapeutic targets. The pronounced conservation of GSK3 observed in *L.* (*V*.) *guyanensis* is consistent with previous studies demonstrating its functional indispensability in *Leishmania* species, while also indicating the existence of structural, regulatory, and kinetic differences relative to mammalian homologs that may be exploited to achieve molecular selectivity [54,55].

In addition to GSK3, the identification of a calpain family member, albeit with an intermediate quality score, supports the potential relevance of this class of proteases as complementary drug targets [56]. Parasite calpains have been associated with protein turnover, differentiation, and host–parasite interactions, and the detection of partially conserved domains suggests that these proteins may remain amenable to pharmacological modulation [57]. The shorter alignment length observed for the calpain candidate in *L*. (*V*.) *guyanensis* likely reflects species-specific structural divergence or partial domain conservation, a pattern frequently reported for this protein family in trypanosomatids [58].

Overall, the prioritization profile obtained in this study—based on objective sequence homology criteria, statistical significance, and composite quality score ranking—shows strong concordance with the existing body of literature on validated and emerging drug targets in trypanosomatids. These findings reinforce GSK3 as the principal conserved and exploitable therapeutic target in *L*. (*V*.) *guyanensis* and underscore the effectiveness of structure-guided homology-based screening as a robust framework for rational drug target discovery in *Leishmania* genomes, with potential applicability across multiple species of the genus [54,55,59].

### 4.5. Taxonomic Validation Through polA1 Phylogeny

The phylogenetic analysis was important for validating the genome assembly data of *L.* (*V.*) *guyanensis* (MHOM/BR/75/M4147) because it might explain any discrepancies found during the assembly process [60]. The analysis using TREE-PUZZLE v.5.3 showed a high phylogenetic signal of the *polA1* gene, favoring the construction of a well-supported phylogenetic tree. This molecular target has been used to identify *Leishmania* species [61,62], showing to be a potential candidate to differentiate cryptic species such as *L.* (*V.*) *guyanensis* and *L.* (*V.*) *panamensis*, *Leishmania* species associated with tegumentary leishmaniasis in the Americas. This fact is important because, in the last 24 years, the validation of *Leishmania* species has been a matter of discussion. The main taxonomical problem lies in the initial *Leishmania* taxonomical proposal based mainly on the biological and eco-epidemiological characteristics of the current species [63,64,65].

The molecular marker *polA1* was previously employed to phylogenetically distinguish and group *Leishmania* (*Mundinia*) *martiniquensis* in cases where Multilocus Enzyme Electrophoresis (MEE) analysis was unable to differentiate this species [61]. In this study, the phylogenetic analysis using the *polA1* gene clustered in different clades of cryptic species, some *Leishmania* species from the same complex, such as *Leishmania* (*Leishmania*) *donovani* and *L.* (*L.*) *infantum*; *L.* (*V.*) *guyanensis* and *L.* (*V.*) *panamensis*; *L.* (*V.*) *braziliensis* and *Leishmania* (*Viannia*) *peruviana*. All sequences from *L.* (*V.*) *guyanensis* and *L.* (*V.*) *panamensis* species clustered into different monophyletic clades, unlike studies based on an *hsp70*-based approach that presented problems differentiating these cryptic species [66]. Phylogeny based on the *hsp70* gene in *Leishmania* has been of help with the subgenera assignment and raised some important questions on the validity of some species belonging to the *L.* (*V.*) *guyanensis* complex [67]. The phylogenetic analysis performed herein showed the potential of the *polA1* gene for the *Leishmania* complex validation when compared with the *Leishmania hsp70* gene region. The main pitfall reported in *hsp70*-based protocols is their problems discerning some species from the *Viannia* subgenus since the geographical distribution of the *Leishmania* species in the Americas has favored a high ancestral genetic flux [68].

Even using the Multilocus Sequence Typing approach (MLST), the phylogenetic differentiation between *L.* (*V.*) *guyanensis* and *L.* (*V.*) *panamensis* from the *L.* (*V.*) *guyanensis* complex is still problematic [39]. The advances in the etiological characterization of the *Leishmania* genus have suggested modifications in the taxonomic nomenclature of species [69,70] and the validation of complexes within subgenera [71]. Therefore, DNA-sequence-based strategies are recommended instead of traditional isoenzyme-based methods. In line with this, we adopted a molecular approach based on the *polA1* gene. The phylogenetic species concept is based on creating a monophyletic group with characteristics different from other groups and following a model of parental ancestry [71]. Consequently, the tree topology obtained herein using the *polA1* gene from *Leishmania* species suggests the *L.* (*V.*) *guyanensis* species complex validation. Furthermore, the phylogenetic tree indicated nucleotide identity grades of at least 90% among species from the same subgenus, except the *Leishmania* (*Mundinia*) subgenus (84.7%). This parameter indicates the number of identical sites among clade components. Observing the tree topology, it is evident that *L.* (*M*.) *martiniquensis* showed the highest phylogenetic distance when compared visually with the rest of the species from the *Mundinia* subgenus, therefore, influencing the global nucleotide identity value assessed for this group. Indeed, when removing the *L.* (*V.*) *martiniquensis* from the analysis, the *Mundinia* clade clustered species with a nucleotide identity value of 95%, an increasing rate of more than 10%. This result suggests that *L.* (*V.*) *martiniquensis* might be the most divergent species of the whole *Leishmania* genus.

## 5. Conclusions

The de novo assembly of *L*. (*V*.) *guyanensis* (MHOM/BR/75/M4147) provides a high-quality genomic framework that clarifies chromosomal aneuploidy and genomic plasticity within the *Viannia* subgenus. Comparative orthology highlights extensive evolutionary conservation and lineage-specific genetic expansions, establishing a baseline for understanding distinct biological traits. These data effectively prioritized candidate drug targets—notably from the GSK3 and calpain families—reinforcing the importance of conserved signaling and proteolytic pathways for therapeutic intervention. Furthermore, the *polA1* marker proved essential for species-level validation and taxonomic resolution, while revealing high divergence within the *Leishmania* (*Mundinia*) subgenus. By integrating advanced genomic and phylogenetic workflows, this study establishes a robust foundation to accelerate the development of precise diagnostic tools and targeted treatments for leishmaniasis in the Americas.

## Figures and Tables

**Table 1:** Comparative Analysis of *Leishmania* spp. Genomic Assembly Metrics. The table summarizes key assembly quality parameters, including number of contigs, N50 value, number of scaffolds, presence of gaps, and read coverage, among others. These metrics are used to assess the integrity and completeness of the assemblies, informing the optimal strategy for downstream genomic studies in the *Leishmania* genus.

Assembled Genomes of *Leishmania* spp.								
Species	*L. guyanensis* (M4147)	*L. guyanensis* (M4147)	*L. guyanensis* (LgCL085)	*L. naiffi* (LnCL233)	*L. panamensis* (PSC-1)	*L. braziliensis* (M2904)	*L. infantum* (JPCM5)	*L. major* Friedlin Strain
Contigs	14,097	35	10,308	14,682	—	—	—	36
N50 contigs (Kb *)	4743	1.1	9.6	5.7	—	—	—	—
Scaffolds	14,097	35	2,8	6.53	108	—	—	—
N50 Scaffold (Kb)	4743	1.1	95.4	24.3	674	—	—	—
Nº of Gaps	0	0	1557	3853	553	876	440	—
Average Coverage *	40.85×	27×	56×	36×	30×	—	—	—
Genome Size (Mb *)	31.56	32.5	31.01	30.34	30.69	31.24	31.92	32.8
G + C (%) *	57.44	58	—	—	57.56	57.72	59.58	59.7
Protein Coding Genes	7192	—	8.23	8104	7748	8175	8199	8272
Total Nº Genes	7698	—	8376	8262	7933	8.37	8.26	8341
Reference	Current study	Genbank	[39]	[39]	[16]	[16]	[16])	[19]

* Kb: kilobases; Mb: megabases; G + C (%): Guanine-Cytosine content percentage; N50: sequence length such that 50% of the total assembly is contained in sequences of that length or longer; Average Coverage: mean number of times each nucleotide is sequenced; (—): data not available or not applicable for the specific assembly version. Genomes obtained from the current study are compared with representative strains from GenBank and literature references.

**Table 2:** Candidate genes were prioritized using BLASTp homology analysis against reference drug targets (GSK3 and calpain). The table summarizes standard alignment metrics (pident, alignment length, e-value, and bitscore) and a combined quality score utilized for ranking. This score integrates percentage identity, alignment coverage, statistical significance, and bitscore.

Gene	Target	Pident	Length	E-Value	Bitscore	Target Family	Quality_Score
g7485.t1_1	GSK3	92,958	355	0	701	GSK3	0.968310746478873
g2584.t1_1	GSK3	41,176	68	2.92 × 10^−10^	50.1	GSK3	0.63774951944162
g7339.t1_1	GSK3	52,593	135	1.57 × 10^−41^	144	GSK3	0.588149574074074
g7477.t1_1	Calpain	38,889	72	3.43 × 10^−10^	52	CALPAIN	0.569850062043589
g2667.t1_1	GSK3	35,135	111	9.01 × 10^−15^	62	GSK3	0.568017545045045
g3420.t1_1	GSK3	35,294	102	1.22 × 10^−13^	59.7	GSK3	0.564705470588235
g6383.t1_1	GSK3	46,753	77	4.53 × 10^−14^	64.3	GSK3	0.556492642857143
g6725.t1_1	GSK3	36,905	84	2.53 × 10^−12^	56.2	GSK3	0.517262738095238
g491.t1_1	GSK3	37	100	5.58 × 10^−13^	57.4	GSK3	0.4695
g7453.t1_1	GSK3	35,652	115	3.13 × 10^−11^	54.7	GSK3	0.368260260869565

## Data Availability

Sequence data that support the findings of this study have been deposited in the GenBank database under the accession number GCA_051201275.1. The data is publicly available at https://www.ncbi.nlm.nih.gov/datasets/genome/GCA_051201275.1/ (accessed on 12 January 2026). All additional data supporting the findings of this study are provided within the article and its Supplementary Materials.

## Supplementary Materials

The following supporting information can be downloaded at: https://www.mdpi.com/article/10.3390/pathogens15010124/s1.

## Abbreviations

CL	cutaneous leishmaniasis
ML	mucocutaneous leishmaniasis
MEE	multilocus enzyme electrophoresis
VL	visceral leishmaniasis
ORF	open reading frames
ML	maximum likelihood
3D	three-dimensional
SNP	single nucleotide polymorphisms
