# Supplementary material for: Resequencing and De Novo Assembly of Leishmania (Viannia) guyanensis from Amazon Region: Genome Assessment, Phylogenetic Insights and Therapeutic Targets
Source: Pathogens. 2026 Jan 22;15(1):124. doi: 10.3390/pathogens15010124 (PMC12845118; doi:10.3390/pathogens15010124)
Supplement: Supplementary file 1 [file pathogens-15-00124-s001.zip › Captions for Supplementary Materials.pdf]

S1: “List of 69 *Leishmania* genomes, along with their respective GenBank accession numbers, that were retrieved and used in this study. This set of genomes was used to construct a local genomic database for the comparison and correction of genomic annotations and Open Reading Frames (ORFs) and served as the input data for the assessment of the phylogenetic signal.”

S2: "Phylogenetic signal analysis was performed using TreePuzzle. The center of the triangle indicates 0.6% unresolved taxa, meeting the criterion of presenting less than 30% unresolved samples for a phylogenetic signal to be present. At the tips (or vertices) of the triangle, a higher proportion of well-resolved taxa is observed, ranging between 32.6% and 33.5%, reflecting the differential support among the phylogenetic groupings."

S3: “Summary table and Sankey diagram (Pavian) showing contig assignment via BLASTn and Kraken2. The table presents the distribution of 14,097 (BLASTn) and 12,534 (Kraken2) contigs assigned to the Leishmania genus. The diagram illustrates the taxonomic flow from phylum to species level, highlighting the genomic purity of the assembly. Taxonomic ranks: K–Kingdom, P–Phylum, C–Class, O–Order, F–Family, G–Genus, S–Species..”

S4 “Statistics from the variant calling process and the read mapping against the *Leishmania* (*V.*) *guyanensis* reference genome (GenBank CP103914-CP103949). This includes the total number of variants identified (36,665 SNPs and 8,210 indels) distributed across the 35 chromosomes. Furthermore, this table details the read coverage values per chromosome, which ranged between 41x and 111x.”

S5: “List of genes identified with copy number expansion (orthologs’ copy numbers) within the core genome of each *Leishmania* species analyzed. The table details the 22 expanded genes found specifically in the *L.* (*V.*) *guyanensis* MHOM/BR/75/M4147 strain, along with their functional annotations and corresponding accession identifiers.”
