# Supplementary figures and images for: Resequencing and De Novo Assembly of Leishmania (Viannia) guyanensis from Amazon Region: Genome Assessment, Phylogenetic Insights and Therapeutic Targets
Source: Pathogens. 2026 Jan 22;15(1):124. doi: 10.3390/pathogens15010124 (PMC12845118; doi:10.3390/pathogens15010124)

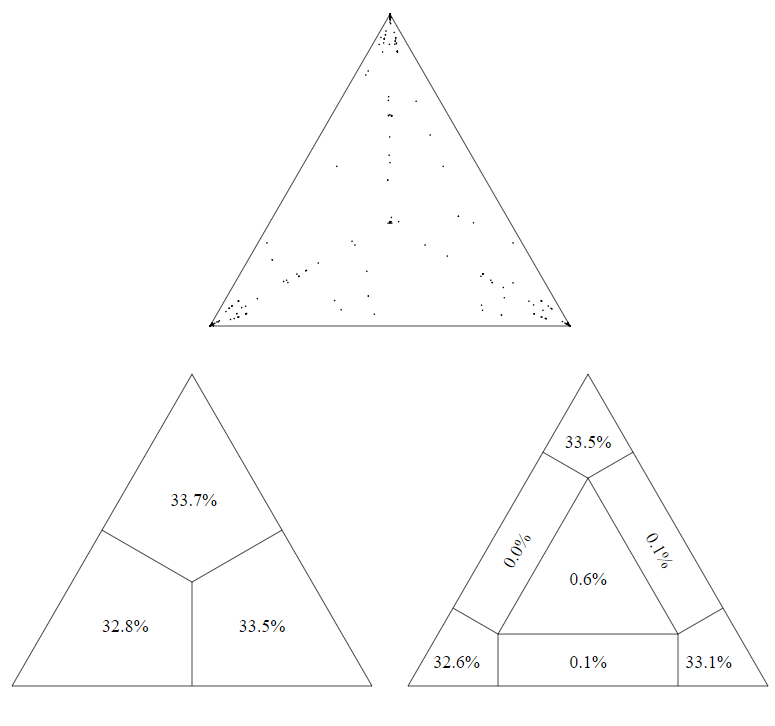

Supplement: Supplementary file 1 [file pathogens-15-00124-s001.zip › S2.png]
